# Kisspeptin Receptor on the Sperm Surface Reflects Epididymal Maturation in the Dog

**DOI:** 10.3390/ijms221810120

**Published:** 2021-09-19

**Authors:** Alessia Gloria, Alberto Contri, Elena Mele, Silvia Fasano, Riccardo Pierantoni, Rosaria Meccariello

**Affiliations:** 1 Faculty of Veterinary Medicine, University of Teramo, Loc. Piano d’Accio, 64100 Teramo, Italy; agloria@unite.it; 2 Faculty of Biosciences and Technologies for Agriculture Food and Environment, University of Teramo, Via Balzarini 1, 64100 Teramo, Italy; 3 Department of Movement Sciences and Wellbeing, Parthenope University of Naples, 80133 Naples, Italy; elena.mele@collaboratore.uniparthenope.it; 4 Department of Experimental Medicine, University of Campania “Luigi Vanvitelli”, 80138 Naples, Italy; silvia.fasano@unicampania.it (S.F.); riccardo.pierantoni@unicampania.it (R.P.)

**Keywords:** Kisspeptin system, Kiss1, Kiss1R, *Canis familiaris*, epididymis, sperm maturation

## Abstract

Alongside the well-known central modulatory role, the Kisspeptin system, comprising Kiss1, its cleavage products (Kisspeptins), and Kisspeptin receptor (Kiss1R), was found to regulate gonadal functions in vertebrates; however, its functional role in the male gamete and its localization during maturation have been poorly understood. The present study analyzed Kisspeptin system in dog testis and spermatozoa recovered from different segments of the epididymis, with focus on Kiss1R on sperm surface alongside the maturation during epididymal transit, demonstrated by modification in sperm kinetic, morphology, and protamination. The proteins Kiss1 and Kiss1R were detected in dog testis. The receptor Kiss1R only was detected in total protein extracts from epididymis spermatozoa, whereas dot blot revealed Kiss1 immunoreactivity in the epidydimal fluid. An increase of the Kiss1R protein on sperm surface along the length of the epididymis, with spermatozoa in the tail showing plasma membrane integrity and Kiss1R protein (*p* < 0.05 vs. epididymis head and body) was observed by flow cytometry and further confirmed by epifluorescence microscopy and Western blot carried on sperm membrane preparations. In parallel, during the transit in the epididymis spermatozoa significantly modified their ability to move and the pattern of motility; a progressive increase in protaminization also occurred. In conclusion, Kisspeptin system was detected in dog testis and spermatozoa. Kiss1R trafficking toward plasma membrane along the length of the epididymis and Kiss1 in epididymal fluid suggested a new functional role of the Kisspeptin system in sperm maturation and storage.

## 1. Introduction

Several studies demonstrated the crucial role of Kisspeptins and their receptor Kiss1R in the regulation of the reproductive axis by stimulating hypothalamic gonadotrophin-releasing hormone (GnRH) release [1,2,3,4,5]. The *Kiss1* gene that encodes the kisspeptin precursor, Kiss1, was discovered in 1996 [6] as a metastasis suppressor in melanoma cell lines, and a few years later, Kiss1R, previously known as GPR54, was discovered [7]. In mammals, the precursor Kiss1 is cleaved into shorter peptides (Kisspeptin (Kp)-54, Kp-13, Kp-14 and Kp-10)), all sharing the C-terminal end and all capable to activate Kiss1R [8,9]. Today, it is well-established that the Kisspeptin system plays a pivotal key role in several aspects of reproduction not only by the direct regulation of GnRH but also by driving the onset of puberty, sex steroid feedback mechanisms [5,10,11], and regulating seasonal reproduction in domestic animals [12,13,14,15], Syrian hamster [16], amphibians [17,18], and some fish [19]. Alongside the central modulatory role, the Kisspeptin system was found to regulate gonadal functions at peripheral in both females [20,21,22] and males [23,24,25,26]. In the testis, the presence of Kiss1 and Kiss1R was detected in Sertoli cells and interstitial cells, while not fully concordant data were found on the presence of Kisspeptin system in germline cells [24,25] (for recent review). Apart from the steroids-secreting Leydig cells, Kisspeptin system is a modulator of the autocrine and paracrine communications that sustains spermatogenesis progression and post-meiotic stages are the main Kisspetin target in the testis of both mammalian and non-mammalian vertebrates [24]. The presence of Kiss1R has been demonstrated in frog, mouse, rat, buffalo, and human spermatozoa [27,28,29], but its functional roles in the male gamete have been poorly understood. Pinto and colleagues characterized Kiss1 and Kiss1R in ejaculated human spermatozoa [29], suggesting an involvement of the Kisspeptin system in the intracellular calcium increase related to capacitation process. Recently, a study confirmed the role of Kisspeptin system in the capacitation process modulating the fertilization capacity of mouse spermatozoa [28].

At the end of spermatogenesis, in the testis, spermatozoa are well morphologically defined but are not functionalized since they are not able to move progressively nor fertilize the oocyte [30]. The maturation of spermatozoa in the epididymis induces functional competence [31,32] and the capability to be motile, acquiring flagellar beat and developing the coordinated axonemal slides, which provide forward propulsion [33]. Furthermore, epididymal maturation induces a modification of sperm proteome (see [30] for a review), such as a remodeling of the plasma membrane lipid composition (see [34] for a review). Little is known about the modification of sperm surface receptors during the epididymal transit, and to the best of the authors’ knowledge, no studies have been conducted to establish the role of the epididymal maturation on the Kisspeptin system in spermatozoa.

Investigations involving human spermatozoa are difficult to approach; thus, most studies have been conducted on rodent models, which differ profoundly in physiology. The dog, for similarities in terms of physiology, disease presentation, and clinical response, appears to be a more suitable model in several fields [35], including epididymal function [36]. The kisspeptin system is conserved in dog and the presence and location of Kiss1 and Kiss1R have been reported in the canine ovary [13], uterus, and trophoblast cells [37]. Furthermore, several Kisspeptin agonists/antagonists have been tested in dog to assess the effects on Kiss1R activation and the outcomes on female reproduction [38,39].

The present study aimed to verify the presence of Kisspeptin system in dog testis and spermatozoa recovered from different segments of the epididymis (head, body, and tail), in parallel with the evaluation of sperm quality parameters. The contribution of every single tract to the configuration of the Kisspeptin system in male mature gametes in the dog as a mammal model is provided.

## 2. Results

### 2.1. Anatomic Attributes of the of Testis and Epididymis

The testicular weight, epididymal weight, and their ratio were 11.9 ± 0.67 g, 3.3 ± 0.25 g, and 0.3 ± 0.01, respectively. In all the cases, no differences were found between left and right organs.

### 2.2. Evaluation of Kiss1 and Kiss1R in Dog Testis, Spermatozoa and Epididymis

#### 2.2.1. Western Blot

**Figure 1:**
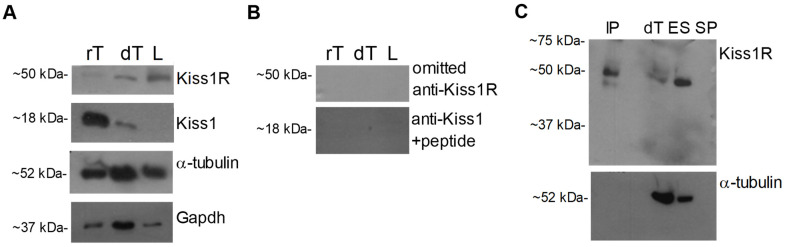
Kiss1R and Kiss1 proteins in dog tissues and Western blot specificity assay. (**A**) Western blot carried out in tissues. (**B**) The specificity of the ~18 kDa Kiss1 band observed in tissues was assayed using the anti Kiss1 antiserum preadsorbed with a five-fold excess of the corresponding antigen. The specificity of the ~50 kDa Kiss1R band was assayed omitting the primary antiserum in Western blot and (**C**) by immunoprecipitation of total proteins (300 µg) extracted from dog testis. dT: dog testis; IP: immunoprecipitation; L: cauda epididymis ligament; rT: rat testis; ES: epididymis sperm (10 µg protein for tract). *N* = 3–4.

The presence of Kiss1R and Kiss1 pro-hormone was evaluated in dog testis by Western blot. Bands of ~50 kDa and~18 kDa were observed in dog and rat testis (positive control), for Kiss1R and Kiss1, respectively. However, Kiss1R but not Kiss1 was observed in the dog cauda epididymis ligament (Figure 1A). Protein loading was assayed by α-tubulin or Gapdh evaluation, revealing bands of the predicted size (~52 kDa and ~37 kDa) in all samples.

Kiss1 signal completely disappeared when Western blot was carried out using the anti Kiss1 primary antiserum pre-adsorbed with a fivefold excess of the corresponding peptide (Figure 1B). Since no blocking peptide was available for Kiss1R antiserum, omission of primary antiserum and immunoprecipitation assays were carried out. The Kiss1R signal of ~50 kDa completely disappeared when Western blot was carried out omitting the incubation with the primary antiserum (Figure 1B). Consistently, the Kiss1R band was observed in dog testis, dog epididymis sperm, and in dog testis immunoprecipitated proteins, but not in the seminal plasma without sperm (negative control), providing evidence of specific binding (Figure 1C). Protein loading was assayed by α-tubulin evaluation, revealing bands of the predicted size in all tissues but not in immunoprecipitated proteins and sperm-free seminal plasma (Figure 1C), as expected.

#### 2.2.2. Kisspeptin System in Dog Sperm and Epididymis Fluid

**Figure 2:**
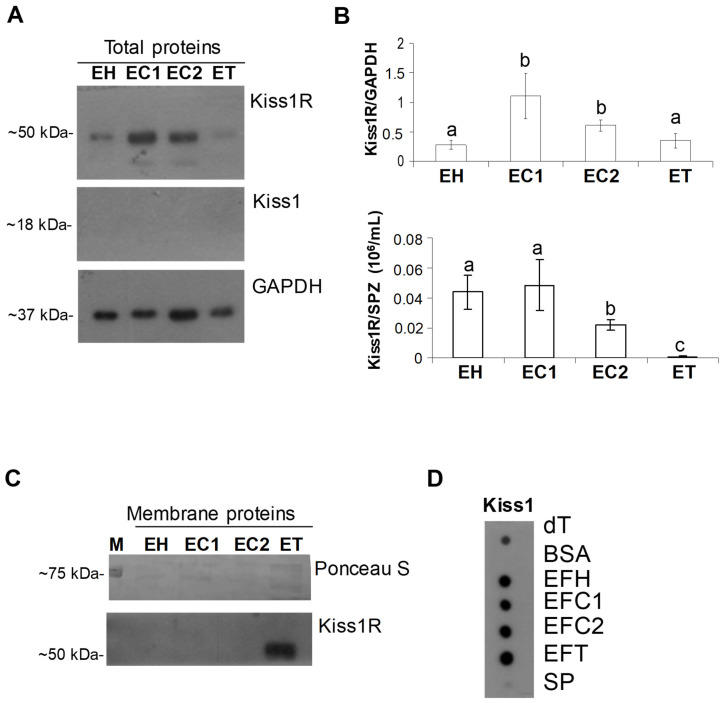
Kisspeptin system in dog sperm and epididymis fluid. (**A**) Representative Western blot of Kiss1R and Kiss1 carried out on total protein extracts from sperm collected at different tracts of the epididymis. (**B**) Protein levels were normalized against GAPDH (upper panel) and then against the SPZ count (lower panel) and data were reported as Kiss1R/GAPDH ± S.D (upper panel) and normalized Kiss1R/SPZ number (10^6^/mL) ± S.D (low panel) (*N* = 4). (**C**) Western blot for Kiss1R carried out on crude membrane fraction (*N* = 3). (**D**) Representative dot blot for Kiss1 carried out on epididymis fluid and spermatozoa-free seminal plasma (*N* = 4). BSA = bovine serum albumin; dT= dog testis, EH: epididymal head sperm; EC1: sperm from the cranial portion of epididymal corpus; EC2: sperm from the caudal portion of the epididymal corpus; ET: epididymal tail sperm; EFH: epididymis fluid from the epididymal head; EFC1: epididymis fluid from the cranial portion of the epididymal corpus; EFC2: epididymis fluid from the caudal portion of the epididymal corpus; EFT: epididymis fluid from the epididymal tail; M = protein ladder; SP: seminal plasma without spermatozoa. Different letters indicate statistically significant differences (*p* < 0.05).

The analysis of Kiss1R and Kiss1 proteins was then carried out on total protein extracts from sperm collected at different tracts of the epididymis, namely the epididymal head (EH), cranial portion of the epididymal corpus (EC1), caudal portion of the epididymal corpus (EC2), and epididymal tail (ET). Western blot revealed the Kiss1R signal, but not Kiss1, in all tracts (Figure 2A) with high levels in EC1 and EC2 (EH vs. EC1 = 0.019; EH vs. EC2 = 0.0017; EC1 vs. ET = 0.029; EC2 vs. ET = 0.017; EC1 vs. EC2 = 0.124) and a constant expression of the receptor in the head and tail of the epididymis (*p* = 0.401) compared to GAPDH used as the internal control (Figure 2B). When Kiss1R signal was normalized against the number of spermatozoa collected in each tract, however, the receptor resulted to be highly expressed in EH and EC1 than in EC2 and ET (*p* < 0.01). Then, to evaluate any possible trafficking of Kiss1R during sperm maturation, crude membrane-enriched protein fractions were prepared from sperm collected at epididymis caput, cranial/caudal corpus, and tail. Western blot revealed the presence of membrane Kiss1R in the fraction of epididymis tail sperm only (Figure 2C), suggesting Kiss1R mobilization during the epididymis transit.

Lastly, the possible secretion of Kiss1 cleavage products in the epididymis fluid was assayed by dot blot revealing immunoreactive spots in all the analyzed tracts and dog testis (positive control) but not in BSA sample (negative control); a very faint signal was observed in the seminal plasma (Figure 2D).

#### 2.2.3. Kiss1R Superficial Localization on Epididymal Spermatozoa

**Figure 3:**
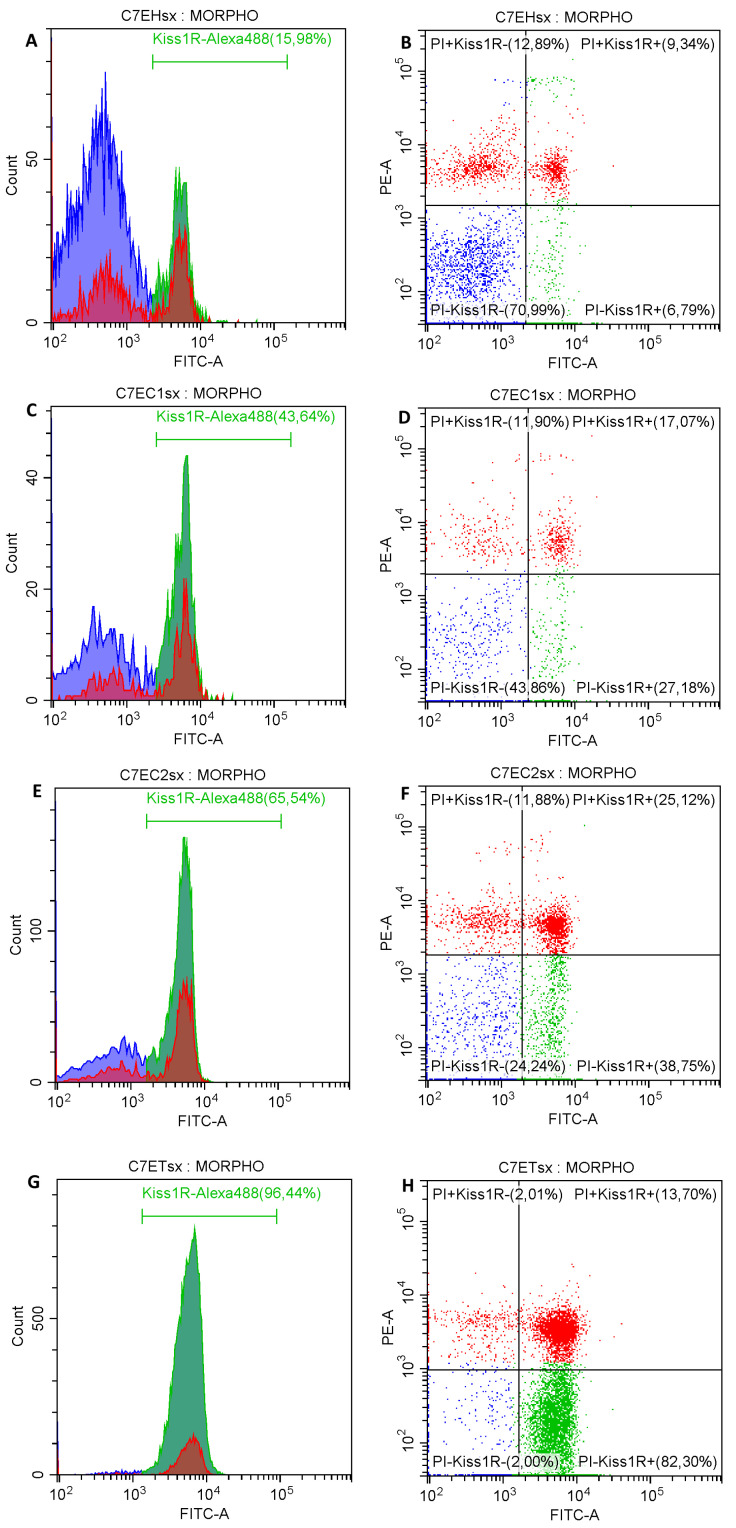
Exemplificative histograms and plots of the Kiss1R surface expression on spermatozoa with membrane integrity in the epididymal head (**A**,**B**, respectively), the cranial portion of the epididymal corpus (**C**,**D**), the caudal portion of the epididymal corpus (**E**,**F**), and epididymal tail (**G**,**H**).

The flow cytometric analysis showed an increase of the Kiss1R expression on sperm surface along the length of the epididymis (Figure 3).

In the EH the proportion of spermatozoa with Kiss1R positivity, both with plasma membrane integrity and damage, was significantly lesser than the other tracts (Table 1; *p* < 0.05). 

In EC1 and EC2 the proportion of spermatozoa with Kiss1R positivity was similar, but an increased proportion of spermatozoa with plasma membrane damage compared with both EH and ET was found (Table 1). In the ET most of the spermatozoa showed plasma membrane integrity and Kiss1R expression compared with all the other tracts of the epididymis (*p* < 0.05). Interestingly, more than 87% of spermatozoa in the ET (both with plasma membrane integrity or damage) exposed Kiss1R on their surface. No differences were found between spermatozoa collected from the right and left epididymis (*p* > 0.05).

**Figure 4:**
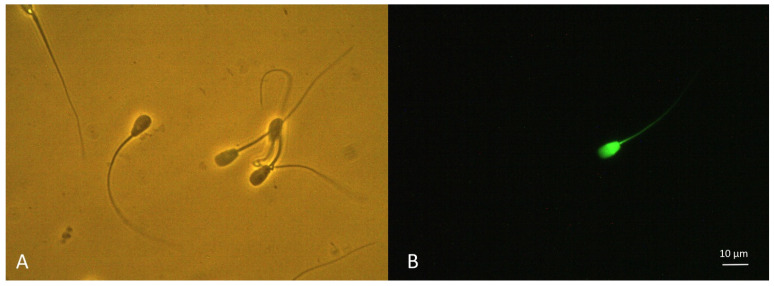
Immunolocalization of Kiss1R on canine spermatozoa recovered from the epididymal head and stained with the rabbit polyclonal antibody against amino acids 141–342 mapping within an internal region of Kiss1R labelled by a Zip Alexa Fluor 488 Rapid Antibody Labelling Kit. On the left (**A**), the field (400× magnification) was acquired using phase contrast microscopy and showed the presence of several spermatozoa. On the right (**B**), the same field was acquired using epifluorescence microscopy (long pass filter; 470–490 excitation, >510 nm emission). Among the spermatozoa present in the field, only one spermatozoon showed a bright green fluorescence, indicating the presence of Kiss1R on sperm surface (bar = 10 μm).

The evaluation of Kiss1R detection in spermatozoa with plasma membrane integrity and damage was confirmed by epifluorescence microscopy (Figure 4). A significant concordance was found between the different subpopulations detected by flow cytometry and the counterpart detected by epifluorescence microscopy (Kiss1R−/PI− vs. eKiss1R−/ePI−: R = 0.842, *p* = 0.002, Kiss1R+/PI− vs. eKiss1R+/ePI−: R = 0.864, *p* < 0.001; Kiss1R+/PI+ vs. eKiss1R+/ePI+: R = 0.814, *p* = 0.028; Kiss1R−/PI+ vs. eKiss1R−/ePI+: R = 0.826, *p* = 0.012).

### 2.3. Sperm Attributes along the Length of the Epididymis

During the epididymal transit spermatozoa significantly modify their ability to move and the pattern of motility (Table 2). 

In the EH, a significant lesser proportion of spermatozoa showed sperm motility, with no spermatozoa classified as progressive. These spermatozoa were less rapid and progressive (Table 2). In the EC1 a significant increase of all kinetic parameters was detected compared with the head (*p* < 0.05). In the caudal portion of the epididymal corpus, an increase in total and progressive motility was detected (*p* < 0.05), as a result of the increased velocity of spermatozoa (VAP, VSL, and VCL; *p* < 0.05), while sperm progressiveness (STR and LIN) were similar. Interestingly, a clear difference in ALH between EC1 and EC2 was found (Table 2; *p* < 0.05). Spermatozoa recovered from the tail of the epididymis showed a higher percentage of motile and progressive spermatozoa, characterized by rapid (VAP, VSL, VCL; *p* < 0.05) and progressive (STR and LIN; *p* < 0.05) kinetic. Only ALH and BCF were similar to the EC2 (Table 2). No differences were found between spermatozoa collected from the right and left epididymis (*p* > 0.05).

The percentage of primary abnormality of spermatozoa remained similar in the different tracts of the epididymis (Table 3, *p* > 0.05). A significant increase of the secondary abnormalities was detected in EC1 and EC2 (*p* < 0.05) compared with both EH and ET, as the result of the increase in midpiece bend. The percentage of this specific abnormality reduced greatly in the ET, where the proportion of secondary abnormalities was similar to the EH (Table 3). A large proportion of spermatozoa in the different tract showed cytoplasmic droplet on the midpiece. Differences were found in the localization of the cytoplasmic droplet, with a proximal localization in all spermatozoa with cytoplasmic droplet collected from the EH, and distal localization in nearly all spermatozoa with cytoplasmic droplet in the ET. The localization of the cytoplasmic droplet seemed change in the epididymal body, as the majority of sperm showed proximal cytoplasmic droplet in the EC1, while in the EC2 the localization of the cytoplasmic droplet was distal (Table 3). No differences were found between spermatozoa collected from the right and left epididymis (*p* > 0.05).

A progressive increase in protaminization was detected during epididymal transit. The proportion of spermatozoa with protamine deficiency, appearing with bright fluorescent yellow color under epifluorescence microscopy, increased progressively and significantly along the length of the epididymis. The lesser value of the protamine deficiency was recorded in the ET (Table 3). Finally, no differences were found between spermatozoa collected from the right and left epididymis (*p* > 0.05).

## 3. Discussion

The Kisspeptin system is an evolutionarily conserved signaling system notably involved in the central control of reproduction, but also expressed at periphery within gonads, gametes, and reproductive tracts in both sexes [20,21,22,23,24,25,26]. In the present study, we preliminarily analyzed the presence of Kiss1 and Kiss1R in the dog testis and then we evaluated the system in parallel to the functional maturation of sperm attributes and storage along the transit in the epididymis.

Present data revealed the presence of both Kiss1 and Kiss1R proteins in dog testis and cauda epididymis ligament, in accordance with the annotation of *Kiss1R* and *Kiss1* genes in dog genome, and the location of both proteins in dog ovary [13], uterus, and trophoblast cells [37]. The presence of the system in the testis in consistent with data from different mammalian and non-mammalian vertebrates that revealed possible role in steroidogenesis, spermatogenesis progression, spermatozoa detachment from Sertoli cells, and sperm physiology [24]. The specificity of Western blot signals was assayed by means of positive controls (i.e., rat testis displayed Kiss1/Kiss1R immunoreactive bands of the same sizes as dog samples [27]), pre-adsorption assay of the anti-Kiss1 antiserum with large amounts of the corresponding antigen, or immunoprecipitation assay of dog testis protein with the anti-Kiss1R antiserum in presence of negative controls (i.e., spermatozoa-free seminal plasma). The presence of the system in the cauda epididymis ligament is quite interesting. This structure could be considered the remnant of a regressed gubernaculum testis, the structure with a pivotal role in the testicular descent, by which the testis-cauda epididymis complex from the original intra-abdominal position descends to the post-natal scrotal position [40]. The simply localization of the Kisspeptin system in this structure, however, give no information regarding a possible role in the mechanism of the testicular descent; thus, further and more specific investigations are required to elucidate this matter.

In this study, Kiss1R, but not Kiss1, protein was observed in spermatozoa collected from the epididymis, suggesting spermatozoa as a target rather than a controller for the Kisspeptin system in epididymal transit. Kiss1R resulted to be present in the total protein extracts from spermatozoa collected in all epididymis tracts, without any significant change in EH or ET, but significant higher levels in the intermediate tracts EC1 and EC2, in parallel to lower percentage of normal spermatozoa. Nevertheless, during the transit within the epididymis, the total amount of Kiss1R decreases in parallel to the increasing number of spermatozoa per tract, the acquisition of appropriate kinematic features and chromatin configuration, and the migration of the cytoplasmic droplet along spermatozoa midpiece. These observations suggest possible Kiss1R intracellular trafficking or degradation. In this respect, ligand-induced desensitization is a critical step in the regulation of Kiss1R activity leading to receptor internalization, sorting for recycling back to the membrane or destruction via lysosome- or proteasome-related pathways [41]. In the present study, data from flow cytometry and immunofluorescence revealed that canine epididymal spermatozoa increased the surface expression of Kiss1R along the length of the epididymis, with the larger amount of the receptor detected on spermatozoa recovered from the tail of the epididymis, particularly in those with intact membrane. In fact, most, but not all the spermatozoa in the epididymal tail showed Kiss1R positivity, linking the requirement of Kiss signaling to the production of functional spermatozoa. The use of crude membrane protein extracts confirmed the presence of Kiss1R only in the membrane of spermatozoa collected from epididymis tail. Thus, Kiss1R trafficking and recycling back to the membrane may occur during spermatozoa transit in the epididymis tracts to ensure the appropriate timing for the acquisition of sperm kinematic and morphological features and the storage in preparation for ejaculation. 

The detection of Kiss1R on spermatozoa recovered from the tail of epididymis was consistent with a previous study in mice [28]. In that study, the expression of Kiss1R was reported on spermatids and spermatozoa in epididymal tail as well as on epididymal epithelium. In mouse developing spermatids, Kiss1R was not detected at the plasma membrane level, but at the acrosomal level [28], suggesting a localized role in the formation of the organelle rather than a functional implication at the cellular level. In the spermatozoa from epididymal tail, Kiss1R was also reported, with localization at the acrosomal level in mouse spermatozoa [28]. Unfortunately, no data were reported regarding the proportion of spermatozoa showing Kiss1R positivity.

Nearly 95% of ejaculated spermatozoa in humans showed the presence of Kiss1R on their surface [29]. No studies were specifically performed on ejaculated canine spermatozoa, but it is unlikely that the addition of seminal plasma, during ejaculation, modify the amount of this receptor on the sperm surface. Thus, the expression on most of the spermatozoa collected at the ET, more than 87%, likely reflects the situation present in ejaculated spermatozoa. To the best of the authors’ knowledge, this is the first study in which the role of the epididymal maturation in surface expression of Kiss1R was investigated. The modulatory role of the epididymis in the expression of surface receptors on spermatozoa received limited attention, despite ejaculated spermatozoa expressing several receptors on their surface, including estrogen [42] and progesterone receptors [43]. As for the Kiss1R in the present study, steroid receptors on the sperm surface seems to be related to a process of maturation along the length of the epididymis. In a previous study on canine spermatozoa, the presence of progesterone receptor was detected on spermatozoa collected from the epididymal tail, while sperm collected from the head and corpus showed a small expression of this receptor and testicular spermatozoa showed no progesterone receptor at all [44]. In our study, the surface expression of Kiss1R showed a similar trend, with the maximal expression of Kiss1R on the surface of spermatozoa collected from the epididymal tail. Functional studies on ejaculated spermatozoa in humans and tail epididymis spermatozoa in mice hypothesized the involvement of the Kisspeptin system in the capacitation, hyperactivation, and acrosome reaction [28,29]. In both species, the stimulation of spermatozoa with Kiss agonists Kp-234 induced the increase of intracellular Ca^2+^, relevant to allow capacitation cascade, and the subsequent change in motility pattern and acrosome exocytosis [45]. Similarly, progesterone stimulation of spermatozoa in vitro resulted in the capacitation of spermatozoa of several species, including humans [46,47,48], primates [49], and dogs [50]. Recent studies strongly suggested that the intracellular increase of Ca^2+^ is likely dependent on extracellular Ca^2+^ influx [51,52] via CatSper channel activation [49,52,53].

The maturative role of the epididymis in animals considered in the present study was demonstrated by the effect of the development of sperm attributes. Motility patterns modify during epididymal transit. Progressive spermatozoa, with increased velocities, were reported in the ET. The evolution of the sperm kinetic as spermatozoa progress in the epididymis was previously described in humans [54], rodents [55,56], and equid species [33]. In the epididymal head, spermatozoa acquire flagellation; in the corpus, the predominant pattern is curvilinear; finally, progressiveness was acquired in the distal regions of the epididymis, mainly in the tail [55]. Similar findings were reported in the present study in the dog, with obvious differences related to the motility pattern in different species and slight differences in the timing of epididymal maturation. The reduced progressiveness detected in the present study in the EC1 and EC2 could reflect the modification of midpiece morphology contextually described. Similar findings were reported in other mammals [33,54,55,56].

Differently from other studies in mammals, the passage along the epididymis negligibly modified sperm primary abnormalities. This finding was not consistent with other studies, in which a reduction of sperm abnormalities was progressively detected during epididymal transit, as the result of an active removal of abnormal cells by phagocytosis, possibly mediated by ubiquitin secreted by epididymal epithelial cells [57]. Sperm morphological evaluation confirmed that the majority of spermatozoa in the epididymis showed the presence of the cytoplasmic droplet, with significant difference in the localization in different tracts. The cytoplasmic droplet, as a cytoplasmic residual, showed a migration proceeding in the epididymal migration in the dog since the percentage of cytoplasmic droplet located at the neck, namely proximal cytoplasmic droplet, and those at the passage between midpiece and principal piece, namely distal cytoplasmic droplet, was the opposite in the head and tail of the epididymis. This finding was consistent with previous studies performed in different species, encouraging the general opinion that the location of the structure is linked to the maturity of spermatozoa. Interestingly, the cytoplasmic droplet may contain lipids, RNAs, enzymes, or lipoproteins, but also elements with Golgi characteristics, that may be incorporated into the spermatic cell, thus contributing to plasma membrane modifications and the acquisition of sperm motility [58].

Epididymal transit resulted in the maturation of DNA structure. Chromomycin A_3_ (CMA3) was previously used as an indirect indicator of protamine compaction of DNA in several species [59,60,61,62]. Chromomycin A_3_ has an affinity for GC-rich minor grooves of DNA, that in mature spermatozoa are masked by the arginine-rich regions of protamines, making these sites inaccessible to the stain. Thus, the binding of CMA3 to DNA, and thus the fluorescence of the stain, is proportional to the degree of compaction and stabilization of the DNA [61]. In canine epididymis, we found a progressive increase of the compaction of the DNA, as the proportion of spermatozoa with positivity to CMA3 decrease during epididymal transit. Our findings confirmed the data reported in bovine, where complete packaging of the DNA was found in spermatozoa collected from the cauda epididymis [61].

Interestingly, a small but relevant proportion of spermatozoa recovered from the epididymal head showed the ability to move and to have mature protaminization, and the proportion of spermatozoa with such characteristics increased in the different tracts of the body, to reach the maximal percentage in the ET. This finding underlines that not all spermatozoa in the epididymis matured at the same time [63], and some of them could be considered mature also in the epididymal head. The hypothesis corroborates previous studies in our [33] and other laboratories [44].

Collectively, these results regarding modification of sperm kinetic pattern, sperm morphology, and sperm DNA compaction confirm the maturative role of the epididymis in canine species. The increased proportion of spermatozoa exhibiting Kiss1R in the ET suggests that only normal and mature spermatozoa express a proper receptorial configuration on their surface. In this respect, the present data strongly candidate Kiss1R as a physiological marker of sperm maturity. Further studies in both physiological and pathological conditions are, however, necessary in parallel to the experimental modulation of the system by Kiss1R specific agonists and antagonists and deeper localization analysis.

Since the presence of Kiss1R in spermatozoa and its trafficking during the epididymis maturation, an intriguing question concerns the presence of the ligand. Dog spermatozoa do not express Kiss1 (present data); thus, autocrine, but not paracrine, modulations have to be excluded. In this respect, the epididymal luminal environment has a key role in sperm maturation and storage providing in each segment a microenvironment specifically rich in proteins, ions, and non-coding RNA that, all together, contribute to the regionalized functionality of the epididymis [64].

Indirect evidence raised the possibility of kisspeptin secretion from rodent testis [65] and antioxidant properties of Kp-10 were investigated for the preservation of sperm functions [66,67]. Recently, Zhou and coworkers measured kisspeptin levels in the seminal plasma of 660 Chinese students, revealing higher levels than plasma and a positive correlation to sperm quality parameters [68]. In the wake of these finding, we used dot blot to investigate the possible presence of any Kiss1 products in different tracts of the epididymis. Immunoreactive spots for Kiss1 were reported in all the analyzed epididymal tracts and in dog testis used as positive control, but not in BSA used as a negative control. The Kiss1 antiserum used in this study is directed against the peptide mapping at the C-terminus of Kiss1 of human origin, thus being capable of recognizing both Kiss1 precursor and its cleavage active peptides. Currently, both Kp-10 and Kiss1 precursor Kp-145 have been found in mouse epididymis cauda, corpus, and tail by immunohistochemistry [28], providing evidence that the epididymis has the ability to produce Kiss1 precursors and its cleavage active peptides. Then, these molecules may be secreted in the epididymal fluid to act as paracrine factor for spermatozoa that express the receptors. Further investigations are required in the field to better characterize the specific peptide, the source of Kisspeptin in the epididymal fluid (i.e., testis or epididymal epithelium or both) and the possible involvement of kisspeptin signaling in mammalian capacitation and fertilization. 

In conclusion, Kisspeptin system was detected in dog testis and spermatozoa, confirming the requirement of intratesticular Kisspeptin system to gain sperm production. Furthermore, for the first time in vertebrates, intracellular trafficking of Kiss1R toward plasma membrane in parallel to sperm maturation during epididymal transit was reported. Taking into account the possible presence of Kisspeptins in epididymal fluid, the present data suggest a new functional role of the Kisspeptin system in the maturation and storage of spermatozoa at epididymal level in preparation for ejaculation.

## 4. Material and Methods

### 4.1. Animals

The study was performed on adult half-breed dogs presented at the Veterinary Teaching Hospital of the University of Teramo for routine castration. Due to the great variability in size and age of the dogs presented at the Veterinary Teaching Hospital, the population included in this study was selected in the range of 18–28 kg for the body weight and between 2 and 6 years of age. At presentation, a preliminary clinical examination was performed, followed by ultrasonographic evaluation of the reproductive organs. Furthermore, a complete blood count and blood biochemistry were also performed. Only males in health without clinical, hematological, or ultrasonographic alterations of the reproductive organs were included in the study and submitted to the surgical procedures. The procedures performed in this study were carried out according to the Italian legislation concerning animal care (DL n. 116, 27 January 1992). Furthermore, informed consent for the inclusion of the animals in the trial was obtained by the owner.

### 4.2. Surgical Orchiectomy

The subjects were kept off feed and water for 12 h before inducing anesthesia. All dogs were pre-medicated with a combination of 0.2 µg/kg IM dexmedetomidine (Dexdomitor, Vetoquinol Italia, Forlì-Cesena, Italy) and 0.2 mg/kg IM methadone (Semfortan, Eurovet Animal Health, Bladel, the Netherlands). The scrotal area was scrubbed using 4% chlorhexidine scrub solution and the neck of the scrotum was shaved. Pharmacological induction was obtained with 1 mg/Kg propofol (Proposure, Merial Italia, Milan, Italy) IV to effect, and general inhalation anesthesia was maintained with isoflurane 2% (Isoflo, Esteve, Barcelona, Spain). Pain control was performed during the surgical procedures with a constant rate infusion of 10 µg/kg/min fentanyl (Fentadon, Eurovet Animal Health). Neither intrafunicolar nor intratesticular anesthesia was performed. All the dogs underwent open orchiectomy using a pre-scrotal surgical approach. After surgery, each dog received postoperative broad-spectrum antimicrobial therapy for 6 days.

### 4.3. Epididymis Collection and Samples Harvest

After orchiectomy, each testis was dissected and a piece of parenchymal testis and the cauda epididymis ligament was frozen in liquid nitrogen and stored at −80 °C until used for Western blot. The epididymis and part of the deferent duct were carefully dissected from the testis. The testicular vessels were then removed and each testis (right and left) was weighted using a precision balance (Explorer—Ohaus, Parsippany, NY, USA). Then individual epididymis (right and left) was carefully isolated from the major blood vessels and connective tissue, weighted using the precision balance, and transferred in a Petri dish. Each epididymis was divided into four portions, EH, EC1, EC2, and ET, each transferred in individual and identified Petri dishes. Each portion of the epididymis was sliced with a scalpel, and spermatozoa were released in 1 mL of TRIS solution [69] for 60 min at 20 °C. Medium with spermatozoa was then transferred into 1.5 mL tubes and maintained at 37 °C in a water bath. An aliquot of every sample (250 µL) was transferred in a cryovial, plugged directly in liquid nitrogen for 5 min, and stored at −80 °C until the evaluation of Kiss1 and Kiss1R expression. The remaining sample was used for sperm evaluation.

### 4.4. Evaluation of KISS1 and Kiss1R in Dog Testis, Spermatozoa and Epididymis

#### 4.4.1. Total Protein Extraction 

Protein extracts were prepared from dog and rat testes (positive control [27]) and epididymis sperm. Testes were gently homogenized in RIPA Lysis Buffer System buffer (Santa Cruz Biotechnology, Inc., Dallas, TX, USA, sc-24948) following the manufacturer’s instructions. In brief, tissues were gently homogenized in chilled RIPA buffer (1 g tissue/3 mL RIPA) supplied with protease inhibitors cocktail (10 µL/mL RIPA), 200 mM phenylmethylsulfonyl fluoride (PMSF, 10 µL/mL RIPA), 100 mM sodium orthovanadate (10 µL/mL RIPA). Sperm collected from different tracts of dog epididymis (EH, EC1, EC2 and ET) as reported above were pulled down by centrifugation at 800× *g* at 4 °C for 15 min. Sperm pellet was resuspended in ice-cold RIPA buffer supplied with protease inhibitors, PMSF, and sodium orthovanadate as reported above and cells were lysed by sonication. The lysates containing testis and sperm protein were incubated on ice for 30 min. Cleared protein extracts were collected after centrifugation at 11,000× *g* for 30 min at 4 °C. The protein concentration was determined using the Lowry assay [70].

#### 4.4.2. Sperm Membrane Preparation 

Crude membrane enriched protein fraction was prepared from sperm collected from EH, EC1, EC2, and ET as previously reported [71]. Sperm pellets, prepared as reported above, were resuspended in hypotonic buffer (2 mM Tris (pH 7.2), 12 mM NaCl) with protease inhibitors cocktail (10 µL/mL) and 1 mM PMSF. Resuspended pellets were sonicated six times for 15 s on ice, with each pulse separated by 1 min on ice; each time, cells were homogenized by pipetting up and down. Cell debris and tails were pulled down by low-speed centrifugation at 2500× *g* for 15 min. The supernatant containing crude sperm membranes were collected, the protein amount was quantified by Lowry methods [70], and then samples were processed for Western blot.

#### 4.4.3. Western Blot Analysis

Sodium dodecyl sulfate-polyacrylamide gel 10% electrophoresis (SDS-PAGE)-separated proteins (40 µg) were electrophoretically transferred to a polyvinylidene fluoride (PVDF) membrane by TransBlot Turbo Transfer System (Bio-Rad, Milan, Italy). To avoid nonspecific binding, membranes were soaked for 1 h in blocking solution (5% non-fat powdered milk in 1 × Tris Buffer Saline (TBS, 10 mM Tris, 150 mM NaCl, pH 7.5)). Then, filters were incubated with the primary antibody (anti-Kiss1R (Santa Cruz Biotechnology, sc-134499) diluted 1:1000; anti-Kiss1, diluted 1:1000 (Santa Cruz Biotechnology, sc-18134)) in TBS 3% non-fat powdered milk solution overnight at 4 °C on an orbital shaker. After washing three times in TBS/0.25% polyxyethylenesorbitan monolaurate (TBS-T), membranes were incubated with a secondary horseradish peroxidase conjugated anti-rabbit/anti-goat IgG diluted 1:1000 (ImmunoReagents Inc., Raleigh, NC, USA) in TBS 3% non-fat powdered milk solution for 1 h at room temperature (RT) and then washed again in TBS-T. The immune complexes were detected using the Western blotting luminol reagent (Santa Cruz Biotechnology, sc-2048). Then, the membranes, stripped at RT for 30 min in stripping buffer (Santa Cruz Biotechnology, sc-281698), were reprobed with the mouse monoclonal anti-α-tubulin (Elabscience, Houston, TX, USA; E-AB-20036) or anti-Glyceraldehyde-3-phosphate dehydrogenase (GADPH) monoclonal antibody (Sigma-Aldrich, St. Louis, MI, USA; c-8795) diluted 1:7500 and 1:20,000, respectively.

#### 4.4.4. Antisera Specificity Assay

Goat polyclonal anti-Kiss1 raised against a peptide mapping at the C-terminus of Kiss1 of human origin (Santa Cruz Biotechnology, sc-18134) was used for Western blot and dot blot. Specificity assay for Kiss1 antiserum included the use of antibody previously preabsorbed overnight at 4 °C on an orbital shaker with a fivefold excess of the corresponding blocking peptide (Santa Cruz Biotechnology, sc-18134 P) in 500 mL of PBS (pH 7.5) or the omission of primary antiserum in Western blot.

Rabbit polyclonal antibody raised against amino acids 141–342 mapping within an internal region of Kiss1R (Santa Cruz Biotechnology) and sharing 92% identity with dog Kiss1R was used for morphological analyses and Western blot. Since any blocking peptide is available for this antiserum, specificity was assayed by Western blot carried out omitting the incubation of primary antiserum and by immunopreciptation assay as previously reported [27]. In brief, 300 µg of dog testis protein were first diluted in RIPA buffer in a final volume of 500 µL and then incubated for 1 h at 4 °C with 1 µg of primary antiserum. After that, 20 µL of Protein-G plus agarose (Santa Cruz Biotechnology, sc-2002) were added and tubes were incubated overnight at 4 °C on a rotating platform. Then, immunoprecipitate was collected by centrifugation at 1000× *g* for 5 min at 4 °C and washed three times in PBS, each time repeating the centrifugation step. The last pellet was resuspended in 40 µL of Laemmli buffer (Bio-Rad), boiled for 5 min, and processed for SDS-PAGE and Western blot.

#### 4.4.5. Detection of Kiss1R on Surface of Epididymal Spermatozoa

Kiss1R expression was evaluated using the rabbit polyclonal antibody raised against amino acids 141–342 mapping within an internal region of Kiss1R (Santa Cruz Biotechnology). The antibody was labelled by a Zip Alexa Fluor 488 Rapid Antibody Labelling Kit (Invitrogen, Eugene, OR, USA), following the manufacturer’s instructions. After preparation, the antibody was used at 1:50. The sperm concentration of all samples was standardized at 50 × 10^6^ cell/mL by dilution with TRIS, then 200 µL from each sample was incubated with labelled Kiss1R antibody for 30 min at room temperature. An aliquot of 300 µL of TRIS was then added and the sample was centrifuged at 500× *g* for 5 min. The supernatant was removed and the sample was resuspended in 1000 µL of TRIS buffer. Finally, 2.4 µmol/L (final concentration) of propidium iodide (PI, LIVE/DEAD^®^ Sperm Viability Kit; Molecular Probes, Eugene, OR, USA) were added, incubated for 15 min at room temperature, and evaluated by flow cytometry, as below described. Thus, 4 subpopulations were detected: spermatozoa with green fluorescence were classified Kiss1R positive (Kiss1R+/PI−), those with red fluorescence were classified membrane damaged (Kiss1R−/PI+), those with both the fluorescences were Kiss1R positive and damaged (Kiss1R+/PI+), and those unstained were Kiss1R negative and with membrane integrity (Kiss1R−/PI−).

The evaluation was performed using the CytoFLEX flow cytometer (Beckman Coulter, San Jose, CA, USA), equipped with a 488 nm (50 mW) wavelength laser. For all evaluations, a morphological gate was created in the side scatter (SSC) and forward scatter (FSC) plot to include only spermatozoa. Spectral compensation was performed analyzing non-stained and single-stained spermatozoa. Spermatozoa were acquired with FL1 (525/40 nm) for Alexa Fluor 488 conjugated Kiss1R, and FL3 (610/20 nm) for the PI. Flow cytometric analysis was performed at a flow rate between 300 to 450 events/s, and acquisitions were stopped at 20,000 events.

To confirm the detection of Kiss1R on the sperm surface, 30 randomly selected samples were evaluated contextually with an epifluorescence microscope (BX51, Olympus Lifescience, Tokyo, Japan) equipped with a long pass filter (U-MNIB2, Olympus Lifescience; 470–490 excitation, >510 nm emission long pass). Spermatozoa with green fluorescence were classified as Kiss1R positive (eKiss1R+/ePI−), those with red fluorescence were classified as membrane damaged (eKiss1R−/ePI+), those with both the fluorescence were Kiss1R positive and damaged (eKiss1R+/ePI+), and those unstained were Kiss1R negative and with membrane integrity (eKiss1R−/ePI−). The proportion of these subpopulations were compared with those recorded in the flow cytometric analysis.

#### 4.4.6. Dot Blot Analysis

After sperm pull-down, the aforementioned epididymal collection medium was processed for dot blot as previously reported [72]. In brief, the epididymal protein content was assayed by the Lowry method [70]. Proteins from dog testis, epididymal fluid (10 µg), or Bovine Serum Albumin (BSA, negative control) (10 µg) were applied spotting directly onto a methanol-activated PVDF membrane. Air dry membrane was processed for the detection of Kiss1 as reported above in the Western blot section, but reducing the incubation time at 30 min for blocking and primary antiserum incubation, the latter carried out at room temperature. 

### 4.5. Sperm Quality Evaluation

#### 4.5.1. Sperm Concentration

Sperm concentration was estimated using a Bürker counting chamber (Merck, Leuven, Belgium) after dilution of 1:100 (EH, EC1, and EC2) or 1:1000 (ET) with 0.9% NaCl solution with 3% glutaraldehyde. For each sample, at least 400 spermatozoa in two chambers (replicates) were recorded. The concentration reported was the mean of the two measurements.

#### 4.5.2. Sperm Kinetics

The semen samples were evaluated for sperm kinetics using the computer-assisted sperm analyzer (CASA) system IVOS 12.3 (Hamilton Thorne Biosciences, Beverly, MA, USA), as previously validated in canine semen [73]. The procedure was performed as previously described [74]. Briefly, an aliquot of each sample was rewarmed at 37 °C for 5 min and a 5 µL drop was loaded onto a Mackler chamber (Sefi Medical Instruments, Haifa, Israel) after a dilution at 40 × 10^6^ sperm/mL with TEY extender. The parameters were collected and recorded by the analysis of 12 non-consecutive fields. Analysis was performed on 30 images/field at 60 frames per second, and the anti-collision algorithm was activated. In this study, total motility (TM, %), progressive motility (PM, %), average path velocity (VAP, µm/s), straight-line velocity (VSL, µm/s), curvilinear velocity (VCL, µm/s), the amplitude of lateral head displacement (ALH, µm), beat cross frequency (BCF, Hz), straightness (STR, as VSL/VAP, %), and linearity (LIN, as VSL/VCL, %) were recorded. Spermatozoa with a VAP ≥ 80 µm/s and STR ≥ 75% were considered progressive.

#### 4.5.3. Morphology

Sperm morphology was evaluated using phase-contrast microscopy (Olympus BX51, Olympus Lifescience) at 1000× magnification after fixation with 0.9% NaCl solution with 3% glutaraldehyde as previously proposed [75]. Sperm were classified with primary abnormalities originating in the testis during spermatogenesis, and secondary abnormalities, that originate during epididymal maturation [76]. The percentage of spermatozoa with proximal (localized at the neck of the spermatozoon) and distal (localized at the end of the midpiece, at the annulus level) were also recorded, but were not included in the computation of primary and secondary abnormalities. Percentages of both primary and secondary sperm abnormalities were calculated on at least 300 spermatozoa.

#### 4.5.4. Sperm Protamine Deficiency

Sperm protamine deficiency was assessed using chromomycin A_3_ (CMA3; Sigma-Aldrich) which was a fluorochrome that competed for binding sites at the minor groove of the DNA strand, which were more present in immature and uncompacted DNA.

The samples were diluted with TRIS buffer, incubated for 30 min with 0.25 mg/mL CMA3 (final concentration) at room temperature. Positive control was prepared with 5 mM of dithiothreitol (DTT). Samples were washed twice at 300 g for 5 min and resuspended into TRIS buffer. For evaluation, a 10 μL drop of the sample was transferred on a slide, a 22 × 22 mm coverslip was applied, and the sample was analyzed by an epifluorescence microscope (BX51, Olympus Lifescience), equipped with a UV filter (U-MWBV2, Olympus Lifescience; 400–440 excitation, 475 nm emission) at 400 × magnification. For each sample, two slides were prepared; thus, the evaluations were performed in duplicate. Spermatozoa with bright yellow fluorescence (chromomycin-positive sperm), having a large number of minor grooves, were classified with protamine deficiency, while spermatozoa with dull yellow fluorescence were considered to have normal protaminization. The percentage of protamine-deficient spermatozoa was calculated on at least 300 sperm.

### 4.6. Statistical Analysis

The data reported in the present study were reported as mean ± standard error of the mean (SEM). 

The normality of the data was checked using the Shapiro–Wilk test, while the homogeneity of variance was assessed using Levene’s test. Since seminal attributes were normally distributed and homoscedastic, the general linear model (GLM) based on ANOVA was used for data analysis. The tract (EH, EC1, EC2, and ET) and the side (left and right testis) were considered fixed variables. When appropriate, the Scheffè post hoc analysis was used.

Correlations between subpopulations created on the basis of plasma membrane integrity and Kiss1R surface expression estimated by flow cytometry and epifluorescence microscopy were tested by calculating the Pearson’s correlation coefficient.

Western blot signals were scanned and the protein levels were plotted to perform quantitative densitometry analysis (optic density, OD) of the signals. The values were expressed as the ratio between the protein of interest OD and housekeeping protein OD. A *t*-test was carried out to assess the significance of differences. Differences were considered significant when *p* < 0.05. 

Statistical analysis was performed using the SPSS 17.0 software (SPSS Inc., Chicago, IL, USA).

## Figures and Tables

**Table 1:** Sperm subpopulations (mean ± standard error of the mean—SEM) on the basis of plasma membrane integrity (sperm plasma membrane damage—PI+) and sperm surface Kiss1R expression (positivity to Anti-Kiss1R antibody—Kiss1R+) in the different epididymal tracts of the dog.

	EH (Mean ± SEM)	EC1 (Mean ± SEM)	EC2 (Mean ± SEM)	ET (Mean ± SEM)
Kiss1R-/PI− (%)	54.76 ± 3.48 ^a^	24.37 ± 4.15 ^b^	29.27 ± 4.98 ^b^	10.19 ± 2.97 ^c^
Kiss1R+/PI− (%)	19.11 ± 3.37 ^a^	31.99 ± 4.83 ^b^	31.73 ± 5.78 ^b^	75.37 ± 4.61 ^c^
Kiss1R+/PI+ (%)	15.58 ± 1.25 ^a^	32.98 ± 1.89 ^b^	30.74 ± 2.67 ^b^	11.68 ± 1.38 ^a^
Kiss1R−/PI+ (%)	10.55 ± 1.08 ^a^	10.67 ± 1.81 ^a^	8.27 ± 1.19 ^a^	2.76 ± 1.09 ^b^

Epididymal head—EH, cranial portion of the epididymal corpus—EC1, caudal portion of the epididymal corpus—EC2, epididymal tail—ET. In the same raw, values with different superscripts (^a–c^) differ significantly (*p* < 0.05).

**Table 2:** Sperm count and kinetic (mean ± standard error of the mean—SEM) in the different epididymal tracts of the dog.

	TRACT			
	EH (Mean ± SEM)	EC1 (Mean ± SEM)	EC2 (Mean ± SEM)	ET (Mean ± SEM)
Concentration (×10^6^/mL)	6.4 ± 1.2 ^a^	22.8 ± 4.3 ^b^	27.3 ± 3.9 ^b^	368.6 ± 53.9 ^c^
Total motility (%)	2 ± 0.9 ^a^	22.9 ± 4 ^b^	51.9 ± 6.3 ^c^	83.8 ± 4.8 ^d^
Progressive motility (%)	0	2.8 ± 2.4 ^a^	9.2 ± 1.9 ^a^	39.7 ± 7.1 ^b^
VAP (µm/s)	13 ± 3.8 ^a^	38.4 ± 5.3 ^b^	58.4 ± 5.6 ^c^	99.2 ± 4.4 ^d^
VSL (µm/s)	11.2 ± 3.3 ^a^	24.9 ± 4.4 ^b^	37.7 ± 3.8 ^c^	75.2 ± 5.5 ^d^
VCL (µm/s)	25.4 ± 8.3 ^a^	90.2 ± 10.8 ^b^	136.5 ± 13.7 ^c^	174.5 ± 9.6 ^d^
ALH (µm)	0.6 ± 0.6 ^a^	6.5 ± 1.2 ^b^	9 ± 0.7 ^c^	8.2 ± 0.5 ^c^
BCF (Hz)	16.6 ± 4.7 ^a^	30.9 ± 2.3 ^b^	30.3 ± 2.2 ^b^	35 ± 0.8 ^b^
STR (%)	49.5 ± 11.3 ^a^	61.3 ± 4.8 ^b^	59.6 ± 4.4 ^b^	73.1 ± 3.1 ^c^
LIN (%)	30.8 ± 8.2 ^a^	29.4 ± 2.8 ^a^	27.6 ± 2.3 ^a^	45.3 ± 4.1 ^b^

Total motility—TM, progressive motility—PM, average path velocity—VAP, straight line velocity—VSL, curvilinear velocity—VCL, lateral head displacement—ALH, beat cross frequency—BCF, straightness—STR, linearity—LIN, sperm viability, epididymal head—EH, cranial portion of the epididymal corpus—EC1, caudal portion of the epididymal corpus—EC2, epididymal tail—ET. In the same raw, values with different superscripts (^a–d^) differ significantly (*p* < 0.05).

**Table 3:** Sperm abnormalities and sperm protamine deficiency (indirectly estimated by chromomycin A3) reported as mean ± standard error of the mean (SEM) in the different epididymal tracts of the dog.

	EH (Mean ± SEM)	EC1 (Mean ± SEM)	EC2 (Mean ± SEM)	ET (Mean ± SEM)
Normal spermatozoa	89.9 ± 1.4 ^a^	57.3 ± 2.9 ^b^	59.7 ± 2.9 ^b^	90.1 ± 1.4 ^a^
Primary abnormalities (%)	5.5 ± 1.6 ^a^	6.2 ± 1.8 ^a^	5.9 ± 2.2 ^a^	4.8 ± 1.7 ^a^
Secondary abnormalities (%)	4.6 ± 1.2 ^a^	36.5 ± 3.7 ^b^	34.4 ± 3.5 ^b^	5.2 ± 1.2 ^a^
Proximal cytoplasmic droplet (%)	39.3 ± 2.2 ^a^	20.4 ± 8.6 ^b^	3.5 ± 1.9 ^c^	0.8 ± 0.3 ^d^
Distal cytoplasmic droplet (%)	0	14.3 ± 13.5 ^a^	41.3 ± 8.1 ^b^	27.7 ± 6.8 ^c^
Protamine deficiency (%)	45.4 ± 1.2 ^a^	20.3 ± 1.4 ^b^	14.6 ± 1.1 ^c^	4.2 ± 0.8 ^d^

In the same raw, values with different superscript (^a–d^) differ significantly (*p* < 0.05).

## Data Availability

The data presented in this study are available on reasonable request from the corresponding author. The data are not publicly available due to the unavailability of some owners to share individual information regarding their dogs**.**
